# Whole Animal Automated Platform for Drug Discovery against Multi-Drug Resistant *Staphylococcus aureus*

**DOI:** 10.1371/journal.pone.0089189

**Published:** 2014-02-19

**Authors:** Rajmohan Rajamuthiah, Beth Burgwyn Fuchs, Elamparithi Jayamani, Younghoon Kim, Jonah Larkins-Ford, Annie Conery, Frederick M. Ausubel, Eleftherios Mylonakis

**Affiliations:** 1 Division of Infectious Diseases, Rhode Island Hospital, Alpert Medical School of Brown University, Providence, Rhode Island, United States of America; 2 Massachusetts General Hospital, Harvard Medical School, Boston, Massachusetts, United States of America; 3 Department of Animal Science, Chonbuk National University, Jeonju, Republic of Korea; The Scripps Research Institute and Sorrento Therapeutics, Inc., United States of America

## Abstract

*Staphylococcus aureus,* the leading cause of hospital-acquired infections in the United States, is also pathogenic to the model nematode *Caenorhabditis elegans*. The *C. elegans-S. aureus* infection model was previously carried out on solid agar plates where the bacteriovorous *C. elegans* feeds on a lawn of *S. aureus*. However, agar-based assays are not amenable to large scale screens for antibacterial compounds. We have developed a high throughput liquid screening assay that uses robotic instrumentation to dispense a precise amount of methicillin resistant *S. aureus* (MRSA) and worms in 384-well assay plates, followed by automated microscopy and image analysis. In validation of the liquid assay, an MRSA cell wall defective mutant, MW2Δ*tarO,* which is attenuated for killing in the agar-based assay, was found to be less virulent in the liquid assay. This robust assay with a Z’-factor consistently greater than 0.5 was utilized to screen the Biomol 4 compound library consisting of 640 small molecules with well characterized bioactivities. As proof of principle, 27 of the 30 clinically used antibiotics present in the library conferred increased *C. elegans* survival and were identified as hits in the screen. Surprisingly, the antihelminthic drug closantel was also identified as a hit in the screen. In further studies, we confirmed the anti-staphylococcal activity of closantel against vancomycin-resistant *S. aureus* isolates and other Gram-positive bacteria. The liquid *C. elegans – S. aureus* assay described here allows screening for anti-staphylococcal compounds that are not toxic to the host.

## Introduction

*Staphylococcus aureus* is a human commensal that is a leading cause of hospital and community-acquired infections [1], [2], including bacteremia and sepsis [3], [4]. Staphylococci possess a wide spectrum of virulence factors and have developed many strategies to bypass host defense mechanisms [5], [6]. In addition, the rapid development of *S. aureus* strains resistant to β-lactam antibiotics such as methicillin (MRSA) present challenges in the treatment of staphylococcal infections. The CDC estimates that in 2011, MRSA was responsible for 80,461 life-threatening infections in the United States alone [7]. In fact, the number of yearly deaths from MRSA infection has surpassed that of AIDS [7]. These statistics underscore the urgent need for novel anti-infectives effective against *S. aureus*.

Traditional methods of antimicrobial drug discovery have usually involved *in vitro* screening for antimicrobial activity and then further *in vitro/in vivo* testing of the hits for toxicity followed by Structure Activity Relationships (SAR) analysis [8]. One shortcoming of this method of antibiotic discovery is that many compounds that are lethal to bacteria are also toxic to humans. We present here a model using a whole animal host *Caenorhabditis elegans* for antimicrobial screening that enables simultaneous assessment of the toxicity of the compound on the host as well as the efficacy of the compound against the pathogen. In addition to conventional antibiotics that affect bacterial growth or viability, our whole animal screening model allows the identification of immunomodulatory compounds and compounds that affect pathogen virulence.

The free living nematode *C. elegans* has recently become a popular model organism for studying pathogenesis of many microbes [9], [10], including *S. aureus* [11], [12]. *C. elegans* growing on a lawn of *S. aureus* die within five days, whereas nematodes feeding on non-pathogenic *E. coli*, the normal laboratory food source, or non-pathogenic *Bacillus subtilis,* live approximately 14 days [11], [13]. Importantly, key virulence factors that are important for staphylococcal pathogenesis in the nematode model are also involved in pathogenesis in humans [11]. *C. elegans* are relatively inexpensive to maintain and their use does not raise ethical concerns related to the use of mammals in biological research. A main objective of this study was to develop a *C. elegans*-MRSA liquid infection assay for automated, high throughput screening of small molecule libraries for antibacterial compounds. The screening methodology was subsequently used to identify antimicrobials in the Biomol 4 library of FDA-approved drugs that promote survival of infected worms. Proof of principle is demonstrated by the fact that out of the 30 clinically used antibiotics represented in the library, 27 were identified as hits in the screen (Tables 1 and 2). The method described here can be applied to assays with other pathogens with only slight modifications. Our results confirm the utility of *C. elegans* as a screening platform for antimicrobial drug discovery.

**Table 1:** Classes of compounds from the Biomol 4 library that promote survival of nematodes infected with MRSA.

Category	Number of hits
Antibiotics	27*
Anticancer	10
Antiviral	1
Antifungal	1
Antiarthritic drug	1
Non-steroidal estrogen	1
Antihelminth	1
**Total**	**42** (6.6% hit rate)

*- Z scores>3 for 25 antibiotic hits and 2<Z score<3 for 2 antibiotic hits.

**Table 2:** Antibiotic hits and their corresponding Z scores in the *C. elegans*-MRSA infection assay.

Name	Z score
Cefoperazone acid [39]	3.56
Cefotaxime acid [40]	6.44
Clinafloxacin HCl [41]	7.11
Clindamycin HCl [42]	23.82
Doxycycline HCl [43]	5.43
Enoxacin [44]	2.85
Enrofloxacin [45]	7.12
Fleroxacin [46]	3.51
Gatifloxacin [47]	6.73
Levofloxacin HCl [48]	7.16
Lincomycin [49]	21.13
Linezolid [42]	8.18
Lomefloxacin HCl [50]	7.11
Minocycline HCl [51]	5.18
Nadifloxacin [52]	6.68
Novobiocin Na [53]	30.07
Ofloxacin [54]	7.16
Pazufloxacin [55]	7.04
Pefloxacine mesylate [56]	7.16
Rifampicin [42]	29.85
Rifamycin sv [57]	7.12
Roxithromycin [58]	6.44
Rufloxacin [59]	2.01
Sarafloxacin HCl [54]	6.66
Sparfloxacin [60]	6.57
Tosufloxacin [61]	6.73
Troleandomycin [62]	23.10

## Materials and Methods

### Bacterial and Nematode Strains

The *S. aureus* methicillin resistant strain MW2 BAA-1707 (ATCC, Manassas, VA, USA) was used throughout this study. It is a community-acquired (CA-MRSA) strain, SCC*mec* Type IV, Panton-Valentine Leucocidin (PVL)-Positive that was isolated in 1998 from a female patient in North Dakota (USA) [14]. The *S. aureus* strain VRS1 carries a plasmid encoding the *vanA* gene that confers resistance to vancomycin [15]. The cell wall defective strain MW2Δ*tarO* expresses an inactive, truncated variant of TarO containing only 80 aa [16]. Bacteria were grown at 37°C in tryptic soy broth (TSB, Becton Dickinson and Company, NJ, USA).

The *C. elegans glp-4(bn2);sek-1(km4)* double mutant strain was used throughout this study. Nematodes were maintained at 15°C on a lawn of *E. coli* strain HB101 on 10 cm plates [17]. The *glp-4(bn2)* mutation renders the strain incapable of producing progeny at 25°C [18] and the *sek-1(km4)* mutation enhances sensitivity to various pathogens [19], reducing assay time.

### Compound Library

The Biomol 4 library (http://www.enzolifesciences.com/) is a collection of 640 FDA-approved drugs that were chosen for their chemical and pharmacological diversity. The library was obtained from the Institute of Chemistry and Cell Biology (ICCB) at Harvard Medical School in 384-well plates. For all plates, 0.1 µl of each of the 2 mg/ml compound stocks in DMSO was pin transferred to separate wells. The compounds were screened at a final compound concentration of 2.86 µg/ml.

### Z’-factor

Z’-factor is a measure of the quality of the HTS assay pipeline and it is calculated from the positive and negative control data [20]. Z’-factor = 1-((3σ_p_ +3σ_n_)/|µ_p_-µ_n_|)) where σ_p_ and σ_n_ are the standard deviations of the positive and negative controls respectively and µ_p_ and µ_n_ are the means of the positive and negative controls respectively. A Z’-factor >0.5 indicates a robust assay. For experiments used to determine the Z’-factor of the assay, 1% dimethyl sulfoxide (DMSO) was the negative control and vancomycin hydrochloride (Sigma Aldrich, MO, USA) dissolved in DMSO at a final concentration of 10 µg/ml was the positive control.

### Infection Assay for Compound Screen

*S. aureus* MW2 was grown overnight in TSB under aerobic conditions with agitation at 37°C. To simulate the growth environment of *S. aureus* in a wound abscess, the aerobic culture was shifted to anaerobic growth conditions the next day by seeding a 10 ml TSB culture tube with 100 µl of the aerobic culture, sealing the tube in an air-tight manner, and incubating overnight without agitation at 37°C. It has been shown that anaerobically grown *S. aureus* exhibits a different pattern of virulence gene expression than aerobically grown cultures [21]. Two thousand *glp-4(bn2);sek-1(km4)* worms at the L1 stage were grown at 15°C on SK agar plates with HB101 as the food source for four days until the worms reached the gravid adult stage. Embryos were harvested from adult worms according to a previously described method [22] and the eggs were hatched by incubation in M9 buffer at 15°C for two days. Approximately 4,500 L1 hatchlings were grown on SK-HB101 agar plates for 52 hours at the restrictive temperature of 25°C until animals were sterile young adults. The worms were harvested by gently washing them off the plates with M9 buffer.

The HTS assay was performed using 384-well plates (Corning no. 3712). A Union Biometrica Complex Object Parametric Analyzer and Sorter (COPASBioSort) was used to transfer 15 adult worms to each well of the assay plate. The total volume in each well was 70 µl with the final composition being 70% M9 buffer, 19% Sheath solution (Union Biometrica Part no. 300-5101-000), 10% TSB, and 1% DMSO or compounds dissolved in DMSO. The bacterial concentration was adjusted to a final OD_600_ of 0.04. After 5 days of incubation in a humidified chamber at 25°C, the bacteria and other debris were washed from the wells with a microplate washer, leaving 10 µl of assay volume with worms following the final aspiration step. Finally, 60 µl of 0.9 µM Sytox Orange in M9 was dispensed into each well for a final Sytox concentration of 0.7 µM. The plates were incubated overnight at 25°C in a humidified chamber. The plates were imaged the next day using an Image Xpress Micro automated microscope (Molecular Devices), capturing both transmitted light and TRITC (535 nm excitation, 610 nm emission) fluorescent images with a 2X objective.

### Worm Survival Quantification using CellProfiler and Hit Identification

The transmitted and fluorescent images of worms in 384 well plates obtained using the Image Express Micro microscope were processed with the open source image analysis software CellProfiler (http://www.cellprofiler.org/) using a pipeline of image processing and analysis modules as described previously [23], [24]. The ratio of Sytox worm area to bright field worm area, and the resultant percentage survival data, is calculated by the software for each well of the assay plates. In order to identify the hits, the Z score was calculated from the ratio data. The Z score is defined as the number of standard deviations an observation is separated from the mean; Z = (x−µ)/σ where x is the raw sample score, µ is the mean of the population and σ is the standard deviation of the population. Samples with Z>2σ were considered as hits.

### Antimicrobial Activity Testing

Compounds (10 mg/ml stock solution in DMSO) were tested for antimicrobial activity by broth microdilution, adapted from established protocols [25]. The assay was done in triplicate in 384-well plates. The total volume in each well was 40 µl with the final composition being 50% M9 buffer, 50% TSB. Two-fold serial dilutions were carried out to get compounds in the concentration range 0.78–50 µg/ml. The bacterial concentration was adjusted to an initial OD_600_ of 0.03. After overnight incubation at 37°C, the absorbance was measured to determine antimicrobial activity.

## Results and Discussion

### MRSA-*C. elegans* Liquid Killing Assay for High Throughput Screening

*C. elegans*-*S. aureus* infection models have been used in several studies investigating staphylococcal virulence and pathogenesis, as well as in screens for compounds with antimicrobial activity [11], [12], [26]–[30]. Previous work has demonstrated that *S. aureus* is pathogenic to *C. elegans* and staphylococcal infection in nematodes is characterized by bacterial accumulation that causes intestinal distension [31]. In the original agar-based assay, nematodes were fed on a lawn of pathogenic bacteria to establish the infection and at an appropriate time point, worm survival was assayed by gently probing the nematodes with a platinum loop to determine whether they moved in response to touch. While this method might be suitable for small scale screens, a less laborious approach utilizing automation is necessary for high throughput, large scale screening.

**Figure 1:**
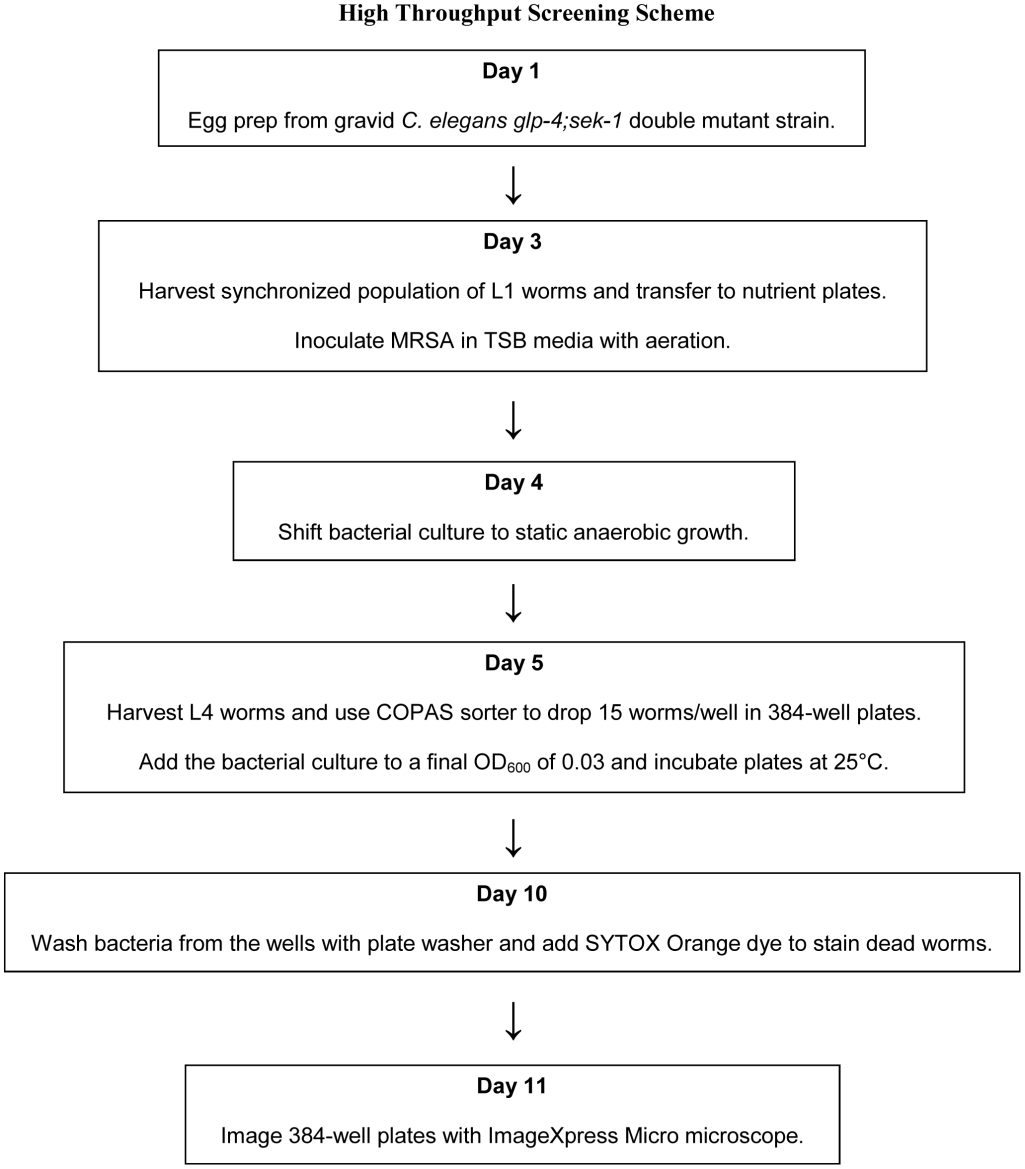
Flowchart representing the time line and work flow of the *C. elegans-*MRSA high throughput screening assay.

**Figure 2:**
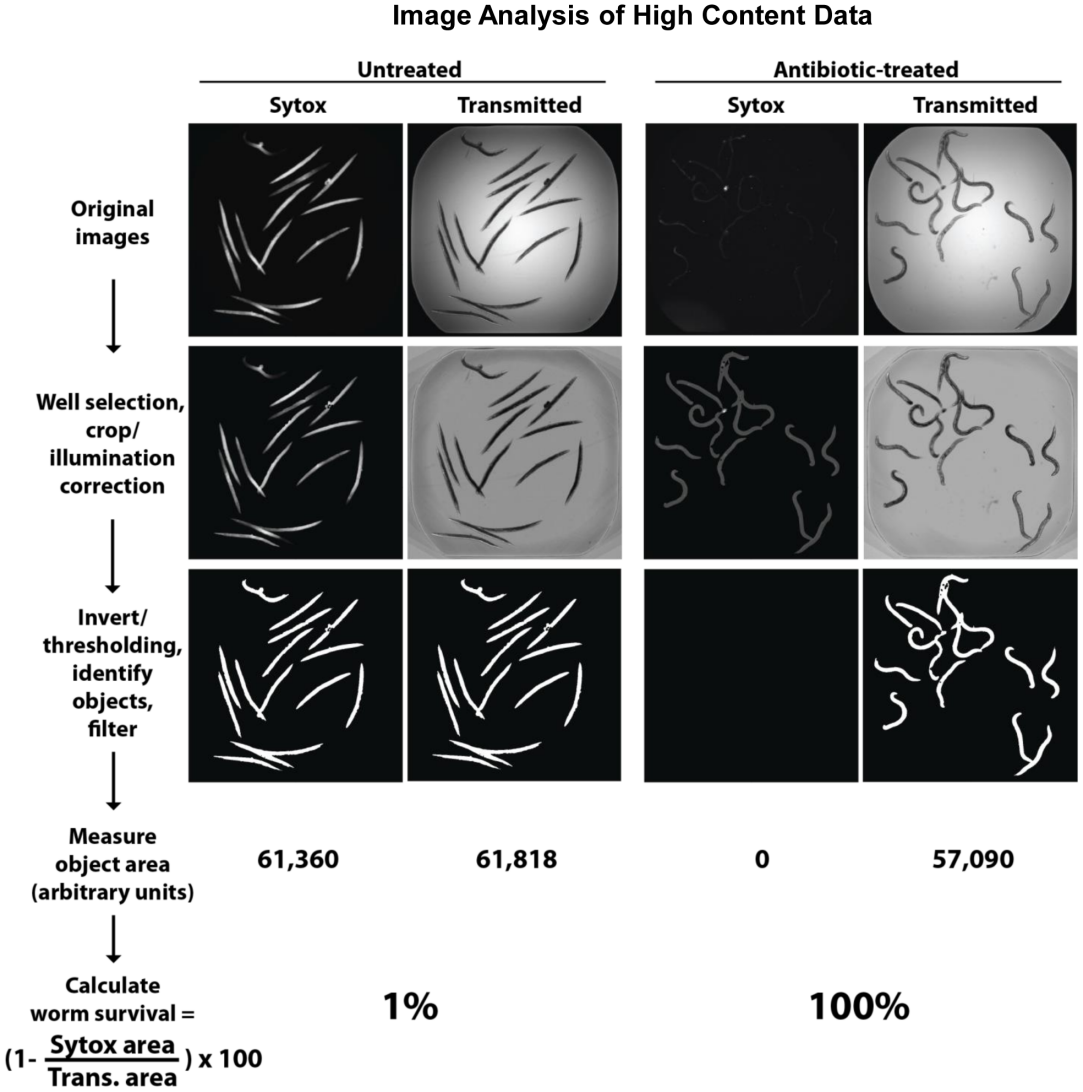
Worm survival quantification using CellProfiler. Worms in 384-well plates were incubated with Sytox Orange, which specifically stains dead worms. The results of several processing steps using CellProfiler are shown. The total area of fluorescent and bright field worms are measured and worm survival in each well is calculated as a percentage. **Top row:** Raw fluorescent Sytox Orange and bright field images of an untreated and an antibiotic-treated well. **Middle row:** Correction for uneven illumination of the bright field images. Cropping of Sytox images so that total fluorescence measurements are only made within worm areas determined by the bright field images. **Bottom row:** Thresholding, identifying worms and filtering for object size.

A liquid-based screening assay was previously established for *C. elegans* infected with *Enterococcus faecalis* [24], [32]. However, adaptations had to be made for the *C. elegans* – MRSA assay. Specifically, in the *C. elegans*-*E. faecalis* HTS assay, larval stage L4 worms were pre-infected with the pathogen prior to sorting. However, using instrumentation to sort and dispense MRSA-infected worms is not feasible because the robotic equipment cannot be efficiently decontaminated after each use. To circumvent this problem, the effectiveness of a co-infection assay, which involved sorting and dispensing the worms in the assay wells and then inoculating the wells with bacteria, was assessed (Fig. 1). Since a standard *C. elegans*-*S. aureus* infection experiment on solid agar is carried out for up to 5 days or longer [11], the liquid assay was carried out for a similar duration. At the end of the assay, the wells were washed to remove the bacteria and worms were stained with Sytox Orange, which preferentially stains dead worms. The assay plates were imaged with an ImageXpress microscope, capturing both transmitted light and TRITC (535 nm excitation, 610 nm emission) fluorescent images with a 2X objective. The use of a 2X objective allows capturing the area of an entire well within one image. The image data were analysed with CellProfiler image analysis software to calculate worm survival based on fluorescence and transmitted light images (Fig. 2). A similar liquid-based screening assay has recently been described for a *C. elegans-P. aeruginosa* pathogenesis model [33].

**Figure 3:**
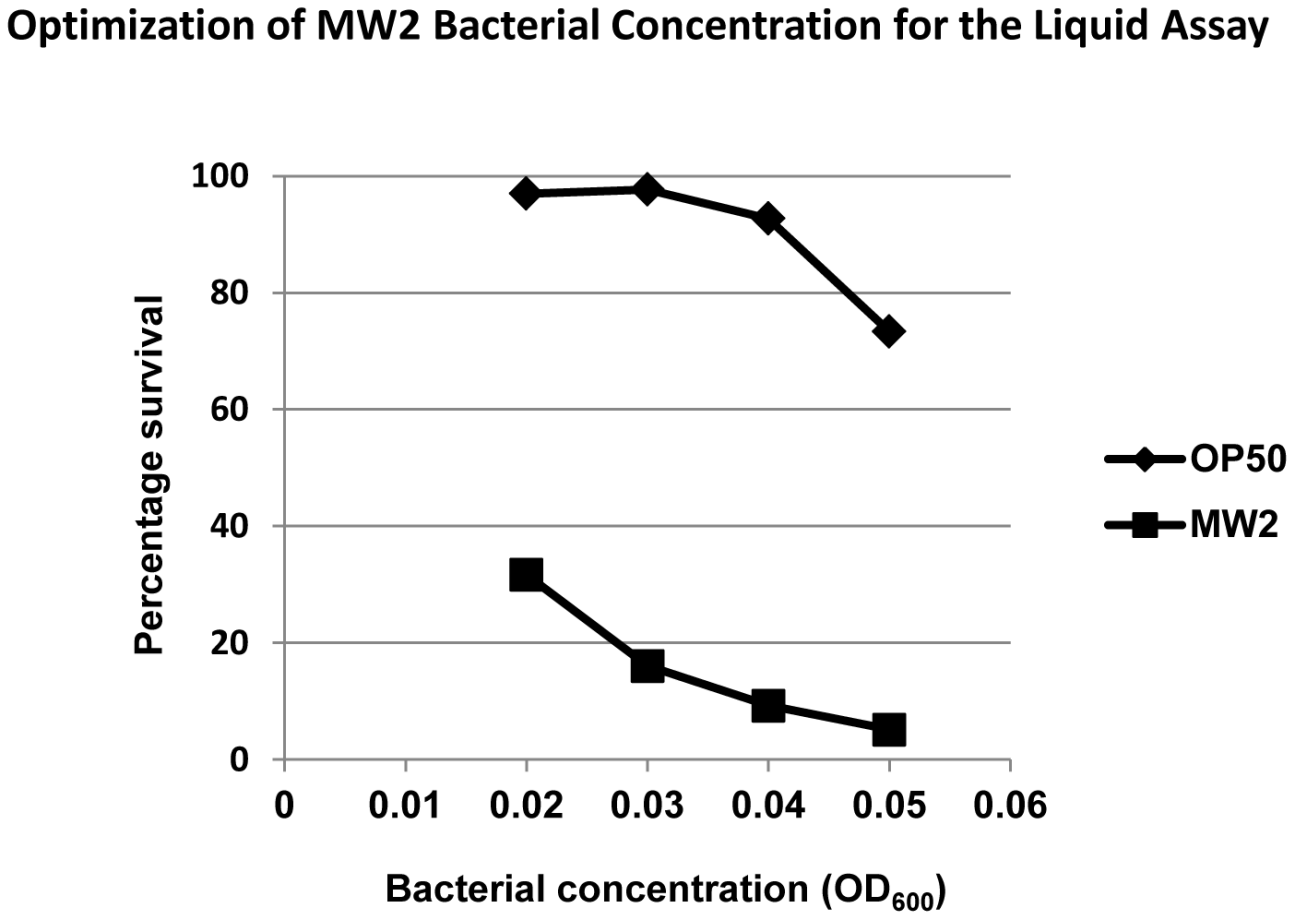
Optimization of starting bacterial concentration used in the infection assay. Worms display a dose dependent susceptibility to the *S. aureus* MW2 starting inoculum.

The liquid *C. elegans–S. aureus* assay was first optimized by testing several concentrations of the MRSA strain MW2 with the starting concentration ranging from an OD_600_ of 0.02 to 0.05. As a negative control, the non-pathogenic *E. coli* strain OP50 was added to the worms at the same concentration while keeping other conditions unchanged. More than 90% of the worms treated with OP50 survived after 5 days of co-infection for initial OD_600_<0.04, but survival dropped to 73% when the starting OD_600_>0.04 (Fig. 3). It is possible that higher bacterial loads might kill worms by suffocation, especially if the bacteria grow at a rate faster than their consumption by the worms. Though *C. elegans* is able to withstand low ambient oxygen levels, prolonged anoxia increases mortality [34]. In contrast to worms exposed to *E. coli*, the survival rate of worms exposed to *S. aureus* MW2 decreased to as low as 5% with an initial OD_600_ of 0.05 (Fig. 3), confirming that *S. aureus* is also capable of killing *C. elegans* in liquid media, similar to assays performed on solid agar plates. Given these results, an OD_600_ of 0.04, was deemed suitable for the infection assay as non-pathogenic *E. coli* OP50 does not cause killing at this bacterial concentration, whereas MW2 causes robust killing.

### Evaluation of the Co-infection Assay

**Figure 4:**
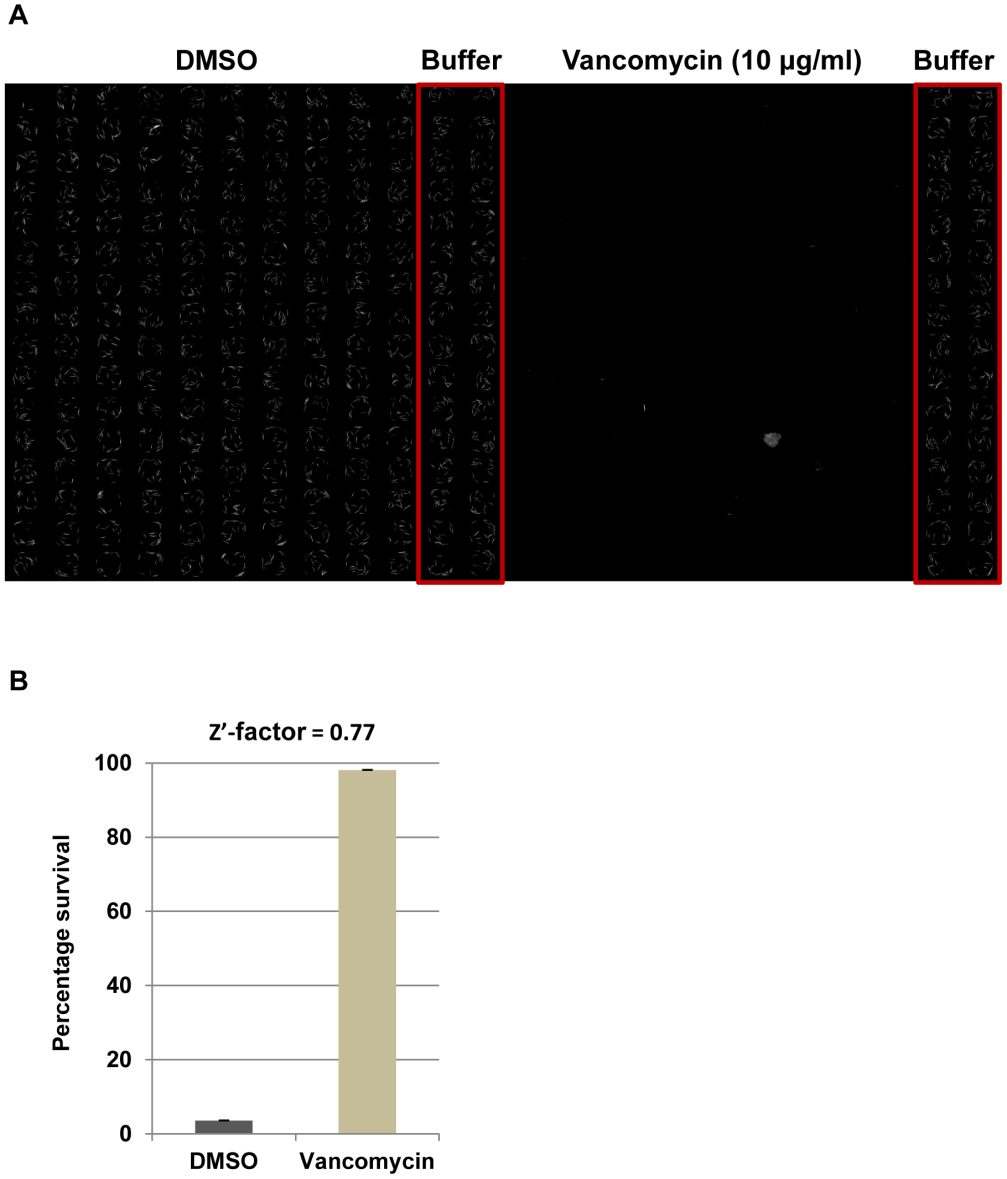
*C. elegans-MRSA* liquid infection assay in 384-well plates. **A)** Assay plates were co-inoculated with nematodes, bacteria and either DMSO (negative control) or vancomycin (10 µg/ml, positive control). The plates were incubated at 25°C for 5 days, washed to remove residual bacteria and imaged. The tiled image was constructed from TRITC fluorescent images of each well from a 384-well plate. **B)** Worm survival was significantly enhanced in wells treated with vancomycin.

In order to determine the reproducibility and reliability of the liquid infection assay, the Z’-factor, a standard measure of robustness of high throughput assays, was determined. The Z’-factor was calculated from CellProfiler-generated percentage survival data from images of wells treated with DMSO (negative control) and vancomycin (positive control) (Figure 4A). The Z’-factor of the screening assay is 0.77 (Fig. 4B), which indicates a very robust assay that is suitable for large scale screening.

### Testing a Cell Wall Defective Strain in the Liquid Infection Assay

**Figure 5:**
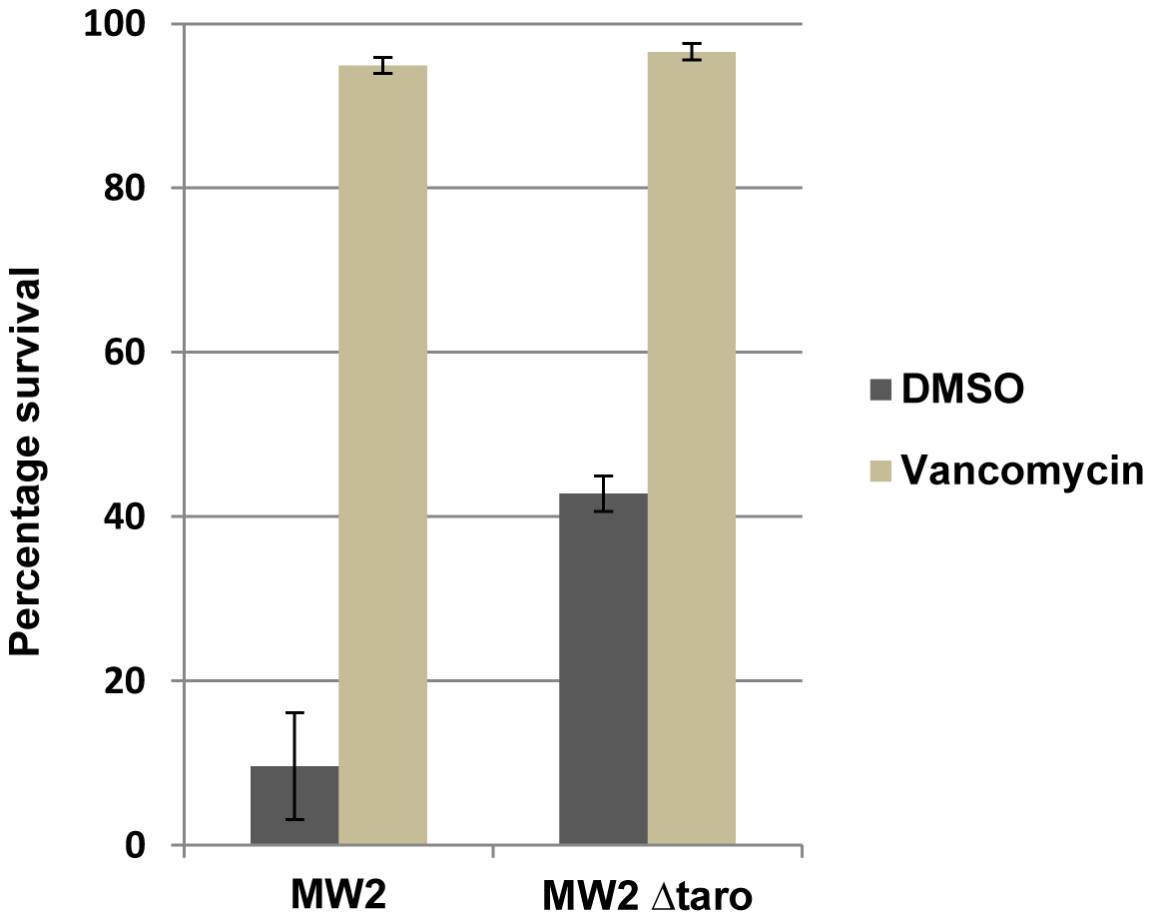
A cell wall defective MRSA strain displays attenuated killing of *C. elegans* in the liquid infection assay. Survival of worms infected with either MW2 or MW2Δ*tarO* was assayed under the same assay conditions. MW2Δ*tarO* was significantly attenuated in killing compared to the wild type MW2 strain in the DMSO wells. Error bars represent standard error.

In order to further confirm that the liquid killing assay behaves similarly to the standard agar killing assay, we compared nematode killing mediated by MW2 and MW2Δ*tarO*, a mutant defective in wall teichoic acid (WTA) biosynthesis [35]. The mutant strain defective in WTA biosynthesis is modestly attenuated compared to a wild type strain in killing *C. elegans* on agar plates [36]. In the liquid assay, worms infected with MW2Δ*tarO* had a higher mean survival rate of 42.8% as compared to 9.6% for worms infected with wild type MW2 (Fig. 5), demonstrating that a previously tested mutant that was less virulent in an agar-based assay is also less virulent in the liquid assay. Although the WTA mutant strain is attenuated in both the agar and liquid-based assays, the degree of attenuation of MW2Δ*tarO* is greater in liquid than on agar. This suggests that the mechanism by which MW2 kills *C. elegans* in liquid may be different than on solid and that WTA biosynthesis may play a greater role in liquid killing than in the agar-based infection assay.

### Identification of Antibiotic Compounds from the Screen

**Figure 6:**
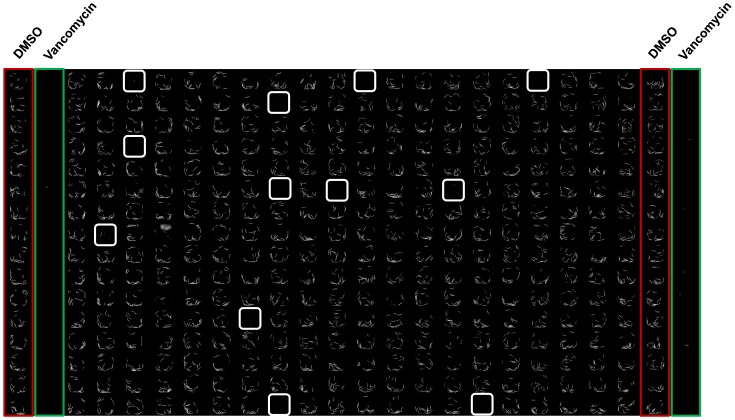
Sytox staining of assay plate. Tiled image of an example assay plate constructed from Sytox fluorescence images. White boxes indicate compounds that enhanced survival of infected worms with Z score greater than 2, the red box indicates DMSO control wells, and the green box indicates vancomycin positive control wells.

Using the optimized liquid screening assay, a pilot screen was conducted with the Biomol 4 compound library consisting of 640 compounds representing several classes of drugs. These compounds include 30 clinically used antibiotics with *in vitro* activity against MRSA [37]–[62]. Based on the Z score threshold of 3, there was a total of 40 hits, 25 of which were known antibiotics (Tables 1 and 2). Antibiotics such as clindamycin, lincomycin, novobiocin, rifampicin and troleandomycin had Z scores greater than 20 suggesting that they are very strong hits. When the Z score threshold was lowered to 2, two more antibiotics, enoxacin and rufloxacin, were identified as hits. Both enoxacin and rufloxacin have *in vitro* activity against MRSA [44], [59], suggesting that the Z score threshold of 2 may be appropriate in identifying hits for this screen. In Figure 6, we present an example assay plate where wells with a Z score greater than 2 have been highlighted in white squares.

Interestingly, the Biomol 4 library includes three different formulations of clindamycin, as a hydrochloride, phosphate and palmitate, but only clindamycin hydrochloride was identified as a hit. Clindamycin hydrochloride is orally administered in capsules whereas the palmitate version is present in oral suspensions and clindamycin phosphate is topically administered. Among the three versions, clindamycin hydrochloride probably had the highest solubility, thereby accounting for its effectiveness in the assay. Thus, the particular formulation of an antibiotic might affect its activity in the assay.

The assay failed to detect three clinical antibiotics, gentamicin, ciprofloxacin and trimethoprim. Trimethoprim is clinically effective on *S. aureus* only in combination with sulfamethoxazole [37]. Gentamicin and ciprofloxacin are mostly active against Gram-negative bacteria and it is clinically recommended to use these antibiotics in combination with vancomycin or rifampicin for treating MRSA infections [63]. Also, we screened all compounds at a relatively low concentration of 2.86 µg/ml, which might not be sufficient for *in vivo* activity since it is likely that compounds may degrade and worms may metabolize and inactivate some of the compounds during the treatment period. Performing the assay at higher compound concentrations might enable a higher rate of detection but it might also increase the possibility that a potential hit might be missed due to toxicity to worms. Ideally, the screen would be performed at varying compound concentrations, which is not practical when screening large chemical libraries.

### Closantel is Active against Vancomycin Resistant Staphylococci

**Figure 7:**
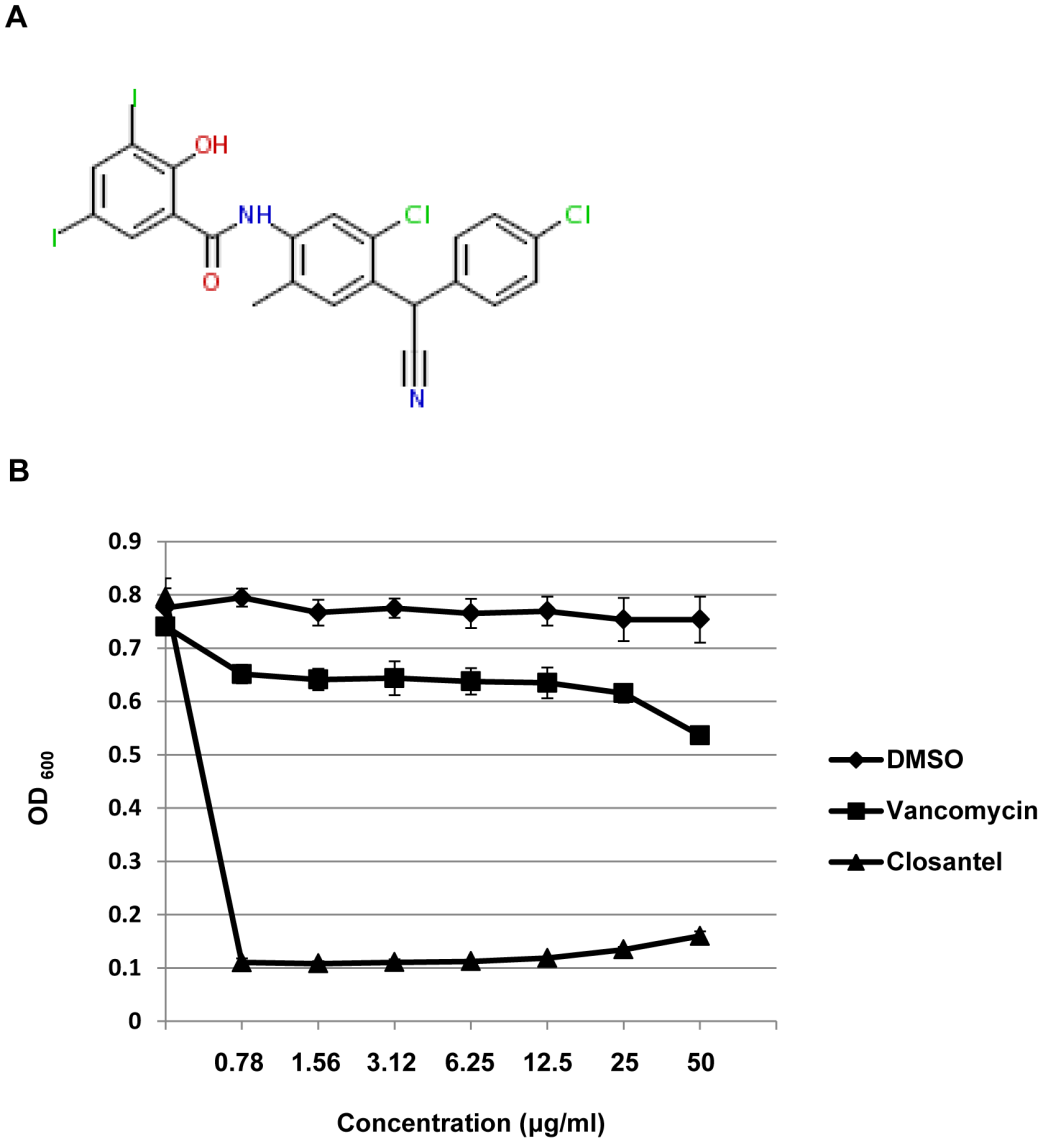
Closantel has a low *in vitro* MIC against VRSA. **A)** Structure of closantel. **B)** *In vitro* antimicrobial activity of closantel was compared with vancomycin against the vancomycin resistant strain VRS1.

One finding from this screen that drew our attention is that the antihelminthic drug closantel (Fig. 7A) was able to prolong the survival of nematodes in the liquid assay. Closantel is marketed as a veterinary antihelminthic drug that is effective against several species of nematodes [64], [65]. Closantel is in the salicylinilide class of drugs and although it has not been well studied, its anthelmintic activity is thought to be as an uncoupler of oxidative phosphorylation [66]. One might have expected that even if closantel does have antibacterial activity, this compound would not have been identified as a hit in our screen because of its toxicity to helminths. However, closantel was identified as a hit with a relatively high Z score of 7.16.

We tested the *in vitro* activity of closantel against other bacteria such as *E. coli*, *B. subtilis*, *E. faecalis* and *Enterococcus faecium* and found that indeed, it has a very low MIC with all Gram-positive bacterial species tested (Table 3), similar to the findings in an earlier study [67]. We found that this compound is also active against another antibiotic-resistant *S. aureus* isolate, the VRSA strain VRS1, with an MIC of at most 0.78 µg/ml (Table 3, Fig. 7B). In comparison, the MIC of oxacillin or vancomycin for the same strain was >256 µg/ml [15]. In order to test whether closantel extends the lifespan of *C. elegans* exposed to MRSA by inhibiting the growth of MRSA in the screening assay, we measured the antibacterial activity of closantel on MRSA in the assay wells in the presence of *C. elegans*. We measured the OD_600_ of the wells with or without closantel at the start and end of an infection assay. Unexpectedly, we found that the OD_600_ of MRSA in wells containing closantel was on average similar to wells without closantel at both the beginning of the assay (OD_600_∼0.03) and at the end of the assay (OD_600_∼0.8) (data not shown). Our data suggest that closantel is not affecting the growth of MRSA in the assay despite the fact that closantel has a low MIC (∼0.78 µg/ml).

**Table 3:** *In vitro* antimicrobial activity of closantel.

MIC (µg/ml)						
Compound	*E. coli* (OP50)	*B. subtilis* (PY79)	MRSA (MW2)	VRSA (VRS1)	*E. faecalis* (MMH594)	*E. faecium* (E007)
Vancomycin	>50	<0.78	3.12	>50	6.25	1.56
Closantel	>50	<0.78	<0.78	<0.78	<0.78	<0.78

Intriguingly, Hlasta et al. [67] showed that closantel inhibits two-component signaling (TCS) regulators in *B. subtilis*. TCS regulators are conserved bacterial transcriptional regulators that control a wide variety of processes in bacteria, such as virulence, antibiotic resistance, and ability to adapt to the external environment [68]–[70]. This suggests that the mechanism by which closantel may promote longer lifespan in *C. elegans* exposed to pathogen is by targeting master *S. aureus* transcriptional regulators. Reasoning that TCS mutants corresponding to closantel targets may be more susceptible to closantel, we tested the activity of closantel on several *S. aureus* strains containing mutations in the VraR-VraS TCS, a system important in promoting antibiotic resistance, and GraR-GraS, a system important for virulence. However, closantel inhibited the growth of the mutant strains to a similar degree as the wild type strain (data not shown). Since it is difficult to interpret these negative results, additional studies are required to determine whether TCS of *S. aureus* is a potential target of closantel. The TCS system, in general, may present an attractive target for antimicrobial therapy as suggested by previous studies [70]–[72].

In addition to the possibility that closantel targets bacterial virulence, it is also possible that closantel could accumulate to low levels in *C. elegans* cells and affect its biology, although it is clearly not toxic to *C. elegans* at the effective dose of 2.86 µg/ml. In contrast, the effective anthelmintic plasma concentrations of closantel in sheep and cattle are ∼50 µg/ml [73]. As stated above, closantel is thought to act as an uncoupler of mitochondrial oxidative phosphorylation, similar to other salicylanilides. It is possible that at the relatively low concentration at which it cures *C. elegans* of an MRSA infection, closantel may not completely disrupt mitochondrial oxidative phosphorylation. In fact, the low concentrations of closantel may be having an entirely opposite effect. Surprisingly, RNAi interference studies in *C. elegans* have shown that slightly reduced function of mitochondrial oxidative phosphorylation machinery extends lifespan in *C. elegans* [74]. As suggested by the RNAi experiments, it is possible that low concentrations of closantel could be having a hormetic lifespan-extending effect on the assay worms.

### Concluding Remarks

In this study, a robust *C. elegans*-based liquid infection assay was designed for testing both the anti-staphylococcal efficacy of compounds and their toxicity to a host in a single step. The ability of the assay to detect all of the clinically relevant antibiotics from a chemical library lends credence to the potency of the assay. Additionally, we report that the agent closantel identified in our screen has significant activity against MRSA and VRSA. Closantel is an attractive candidate for treatment of staphylococcal infections and we are further investigating its mechanism of action and clinical potential. Closantel is a prime example of the possibility of “repurposing” a drug already used in the clinic for other therapies. The assays described here could advance drug discovery using model organisms and decrease the need for mammalian testing.
